# Adult Attention-Deficit/Hyperactivity Disorder and Borderline Personality Disorder: diagnostic and management challenges

**DOI:** 10.1192/j.eurpsy.2024.993

**Published:** 2024-08-27

**Authors:** M. Barbosa, A. R. Fonseca

**Affiliations:** SPSM, Centro Hospitalar de Leiria, Leiria, Portugal

## Abstract

**Introduction:**

Borderline Personality Disorder (BPD) and Attention-Deficit/Hyperactivity Disorder (ADHD), relatively common psychiatric pathologies (5% and 1-2% respectively), share several characteristics, specially impulsivity and emotional dysregulation. With different therapeutic approaches, it is therefore important to distinguish the entities for a correct approach to the patient. Clinical evidence has also demonstrated high comorbidity between two entities, and therefore this recognition is of equal relevance.

**Objectives:**

Analyze the clinical evidence, in order to better understand the dynamics between the two pathologies as comorbid or differential diagnosis, for an appropriate approach to the patient.

**Methods:**

Authors used the Medline database through the Pubmed search engine, with the keywords: “PBP”, “PHDA”.

**Results:**

These two pathologies share impulsive and spontaneous actions with poor thinking about the consequences; nonetheless, ADHD individuals tend to show this impulsivity by being more impatiente when they have to wait, talking over other people, interrupting others; on the contrary, in BPD impulsivity can be showed more as self-harm behaviors.

As for the emotional dysregulation, that both entities share, in the comorbid case it is known that it is the most severe form. This characteristic is part of the central characteristics of BPD where these individuals experience intense and unstable emotions. They have difficulty regulating their emotions which can lead to rapid changes in mood, and they report feelings of emotional emptiness and difficulty in establishing stable relationships. As for ADHD individuals, despite present, it’s not a core symptom, as they have more control over their emotions, and have more adaptative cognitive strategies.

Attention deficit can be a core symptom of a subtype of ADHD and has not yet been reported in patients with PBP, except in comorbid situations. According to studies, 30-60% of patients with PBP report and score on attention deficit scales. Truth is both entities have intelectual disfunctionalities.

Results of genetic studies are very inconsistent, however epigenetic research and reseach focusing on hypothetized vulnerability genes or sites have been promising.

**Conclusions:**

A complete clinical history is particularly important in these cases and sometimes difficult, as so, clinicians should be aware to prevent misdiagnosis and provide the best care for both disorders and the comorbidity. Given that treatment differs between both pathologies, psychotherapy in BPD, and the multimodal approach in ADHD, it is imperative to distinguish the two entities. In comorbid cases, a combination of the two therapies has demonstrated effectiveness but much more studies are needed.

**Disclosure of Interest:**

None Declared

